# High Dose Corticosteroids in Chronic Histiocytic Intervillositis Allowing Prolongation of Pregnancy: A Case Report

**DOI:** 10.1111/jog.70478

**Published:** 2026-09-07

**Authors:** Arti Dave, Victoria Bryant, Manlo Kwok, Rebecca Allen, Alexander Heazell, Kate Wiles

**Affiliations:** ^1^ Barts Health NHS Trust London UK; ^2^ University of Manchester Manchester UK

**Keywords:** chronic histiocytic intervillositis, corticosteroids, placenta

## Abstract

Chronic histiocytic intervillositis (CHI) is a rare placental disorder linked to poor pregnancy outcomes, including fetal growth restriction and perinatal loss. We report a case of recurrent CHI in a 36‐year‐old woman with five previous pregnancy losses. In her sixth pregnancy, placental histology confirmed CHI after preterm delivery. In a subsequent seventh pregnancy, despite early treatment with aspirin, low‐molecular weight heparin, and hydroxychloroquine, she developed uteroplacental dysfunction at 17 weeks with hypertension, proteinuria, and transaminitis. Rescue therapy with high‐dose prednisolone led to maternal clinical improvement, stabilization of fetal Dopplers, and pregnancy prolongation to 30 weeks. Placental histology showed a reduced inflammatory infiltrate compared to the prior pregnancy, suggesting a therapeutic effect from corticosteroids. This case reports the novel use of high‐dose corticosteroid rescue therapy in managing CHI and highlights the importance of early diagnosis, multidisciplinary care, and the need for further research.

## 1 Introduction

Chronic histiocytic intervillositis (CHI) is a rare placental disorder estimated to occur in 1 in 5–10 000 pregnancies [1]. It is a histopathological diagnosis characterized by an inflammatory infiltrate of mononuclear macrophages (histiocytes) within the placental intervillous space, frequently accompanied by perivillous fibrin deposition [2]. The histiocytes are maternal in origin, which suggests a primary immune etiology; which is supported by high rates of co‐existing autoimmune disease [2].

CHI is associated with poor obstetric and neonatal outcomes including fetal growth restriction (FGR) and intrauterine fetal death. The reported perinatal mortality rate for CHI is as high as 55% [3, 4], FGR occurs in 73% of affected pregnancies [3], and 67.4% of pregnancies deliver preterm [4].

CHI has a high recurrence rate of 70%–100% and the optimum management to prevent future adverse outcomes is unclear. Treatment strategies involve immunosuppression and anticoagulant/antiplatelet therapies, though evidence for efficacy is limited. Systematic review data show variable responses to treatments including low‐dose aspirin, low molecular weight heparin (LMWH), prednisolone (typically 10–20 mg daily [1]), hydroxychloroquine, and monoclonal antibodies underscoring the need for further research [5].

## 2 Case Report

A 36‐year‐old female of Black British ethnicity presented in her 6th pregnancy. Her previous pregnancies had been complicated by three second trimester miscarriages at 23, 19, and 20 weeks, a neonatal death after spontaneous vaginal delivery at 24 weeks, and a second neonatal death after an emergency caesarean birth at 33 weeks performed due to severe pre‐eclampsia. Placental histology was not available from any previous pregnancies.

In her 6th pregnancy, she was treated with aspirin 150 mg daily from 9 weeks. Early pregnancy hypertension was managed with labetalol. A history‐indicated cervical cerclage was placed at 18 weeks, and prophylactic‐dose LMWH was initiated at 19 weeks. Serial fetal ultrasound scans took place every 4 weeks from 20 weeks. At 28 weeks, a reduction in fetal growth velocity was noted with estimated fetal weight falling from the 50th to the 10th percentile. There were also signs of early redistribution including a raised umbilical artery pulsatility index (PI) and absent end‐diastolic flow, and a low middle cerebral artery PI. A caesarean birth was performed at 29^+5^ weeks in response to pathological cardiotocography with prolonged fetal heart rate decelerations. Birthweight was 1070 g (0.8th centile), with APGAR scores of 8, 9, and 10 at 0, 5, and 10 min, respectively. Intubation and respiratory support were required initially. The baby was discharged home after a normal cranial ultrasound at the age of 11 days.

**FIGURE 1:**
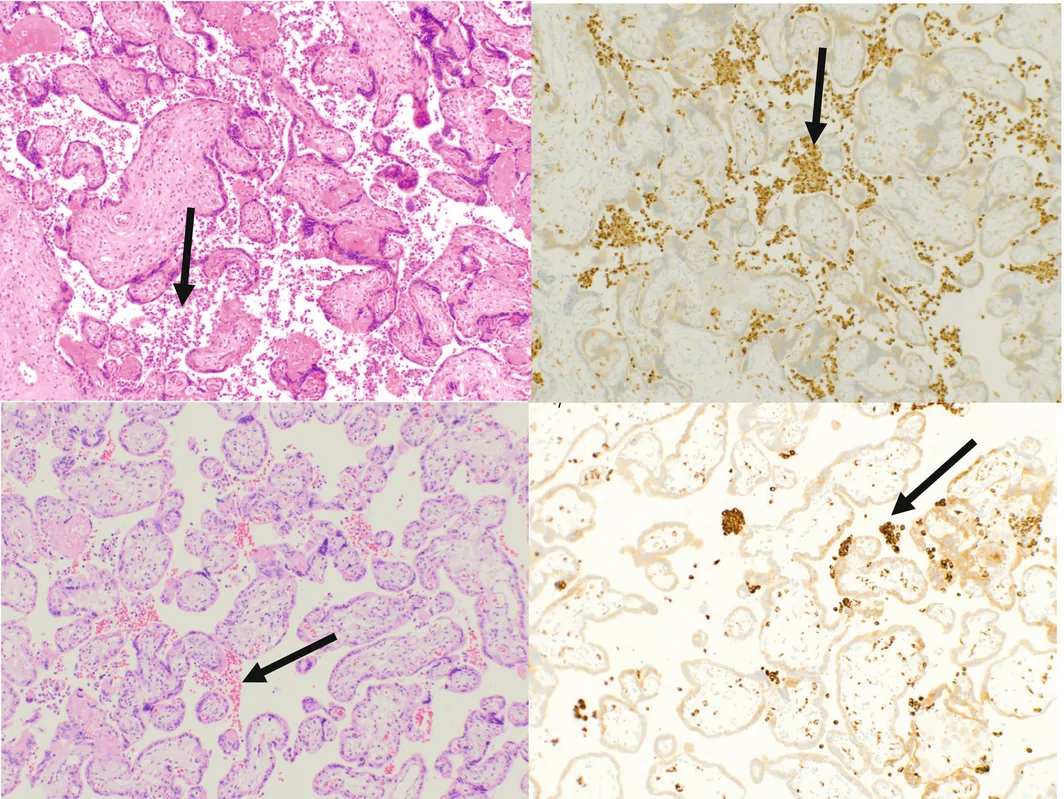
(magnification × 100) Placenta from the 6th pregnancy (top right and top left): A dense infiltrate of histiocytes (arrows) in the intervillous space on H&E (left) and with immunohistochemistry staining for CD68 (right). Placenta from the 7th pregnancy (bottom right and bottom left): Sparse histiocytes (arrows) in the intervillous space on H&E (left) and with immunohistochemistry with CD68 (right).

The trimmed placenta weighed 216 g (25–50th centile for gestation). Histology showed appropriately mature chorionic villi for the gestation with multiple foci of dense histiocytic infiltrate in the intervillous space consistent with CHI (Figure 1). There were no diagnostic features of maternal vascular malperfusion.

Postnatal autoimmune testing detected persistent positive anti‐cardiolipin antibodies; however, measured concentrations were consistently low (IgG 1.5 units/mL, IgM 2.7 units/mL) with negative lupus anticoagulant and beta glycoprotein concentrations. Anti‐nuclear antibodies and rheumatoid factor were not detected. C3, C4, immunoglobulin, corrected calcium, and thyroid‐stimulating hormone concentrations were normal.

The patient was informed of the diagnosis of CHI. She was counseled about the risk of recurrence, including more severe CHI in a future pregnancy, and the importance of contraception to prevent unplanned pregnancy. A plan was made for early initiation of aspirin and LMWH in a future pregnancy. Given her history of cervical incompetence and preterm rupture of membranes, the risks of corticosteroids and other immunosuppression were felt to be high. Hydroxychloroquine was deemed a safer prophylactic option.

She presented in her 7th pregnancy at 10 weeks' gestation; aspirin 150 mg daily, prophylactic‐dose LMWH, and hydroxychloroquine 200 mg daily were commenced. Elevated blood pressure in early pregnancy was managed with amlodipine 5 mg daily with good control. Given her history of mid‐trimester losses, a history‐indicated cervical cerclage was undertaken at 13 weeks supplemented with vaginal progesterone pessaries (Cyclogest) 400 mg twice daily. A urine protein/creatinine ratio (uPCR) at 15 weeks was normal (24 mg/mmol).

At 17 weeks, she was admitted with acutely elevated blood pressure, significant proteinuria (uPCR 412 mg/mmol), and transaminitis (ALT 93 U/L). Serum creatinine was normal (44 μmol/L). Fetal ultrasound revealed an EFW of 164 g (9th centile) with bilateral notching of the uterine arteries and intermittently absent end diastolic flow in the umbilical arteries. A clinical diagnosis of uteroplacental dysfunction due to recurrent CHI was made. She was counseled about the risks to maternal health and the likelihood of a poor neonatal outcome. The option of pregnancy termination was declined.

Following multidisciplinary team discussion, she was commenced on rescue prednisolone 0.5 mg/kg (35 mg) once daily, with dosing extrapolated based on immunosuppressive regimes in other autoimmune disorders. Her blood pressure was controlled with modified‐release nifedipine 30 mg three times daily and labetalol 300 mg three times daily. Following commencement of high‐dose corticosteroids, her transaminitis normalized to an ALT of 18 U/L within 24 days and her urine PCR more than halved from 412 mg/mmol to 143 mg/mmol over the same time (Table 1). Steroid‐induced diabetes was controlled with metformin. With stabilization of blood pressure, kidney function and liver blood tests, the prednisolone dose was weaned by 5 mg/week to a maintenance dose of 10 mg. Serial fetal surveillance showed a thickened and shortened placenta with fetal growth below the 3rd percentile. Although umbilical artery end diastolic flow was restored, umbilical artery and ductus venosus pulsatility indices were high with a positive a wave. Nifedipine was switched to oral hydralazine due to significant gum hypertrophy.

**TABLE 1:** Biochemistry and fetal monitoring in 7th pregnancy before and after high‐dose corticosteroid initiation.

Clinical marker	Early pregnancy (10 weeks)	Before prednisolone 0.5 mg/kg (17 weeks)	After prednisolone 0.5 mg/kg (21 weeks)
BP (mm/Hg)	116/72	168/98	139/80 (treated)
ALT (U/L)	27	93	31
Urine PCR (mg/mmol)	24	412	143
Creatinine (μmol/L)	61	44	46
Fetal doppler		Intermittent absent end‐diastolic flow in the umbilical artery	Normal umbilical artery Doppler

At 30 weeks' gestation, nephrotic‐range proteinuria recurred (uPCR 900 mg/mmol) with maternal hypertension despite four agents. Corticosteroids for fetal lung maturation and magnesium for fetal neuroprotection preceded a Caesarean birth performed due to uncontrollable maternal hypertension in the context of uteroplacental dysfunction. Birthweight was 1209 g (4th centile) with APGAR scores of 1, 8 and 9, at 0, 5 and 10 min. The baby required intubation for 6 days with ongoing care in the neonatal unit for a further 62 days. At 5 months of age, growth and developmental milestones were normal. Hydroxychloroquine was stopped at delivery and prednisolone was weaned over the first 4 post‐partum weeks. Postnatally, maternal chronic hypertension was diagnosed, which was well controlled on two agents at 6 months postpartum, with normal liver and kidney function.

The trimmed placenta weighed 295 g (25–50th centile for gestation). Placental histology showed appropriately mature chorionic villi for the gestation. There was multifocal villous agglutination with syncytial bridges suggestive of incipient infarction. This time only a patchy infiltrate of intervillous histiocytes was present (Figure 1). Compared to the severe histology in her previous (6th) pregnancy, the infiltration of intervillous histiocytes in this pregnancy was sparse.

**FIGURE 2:**
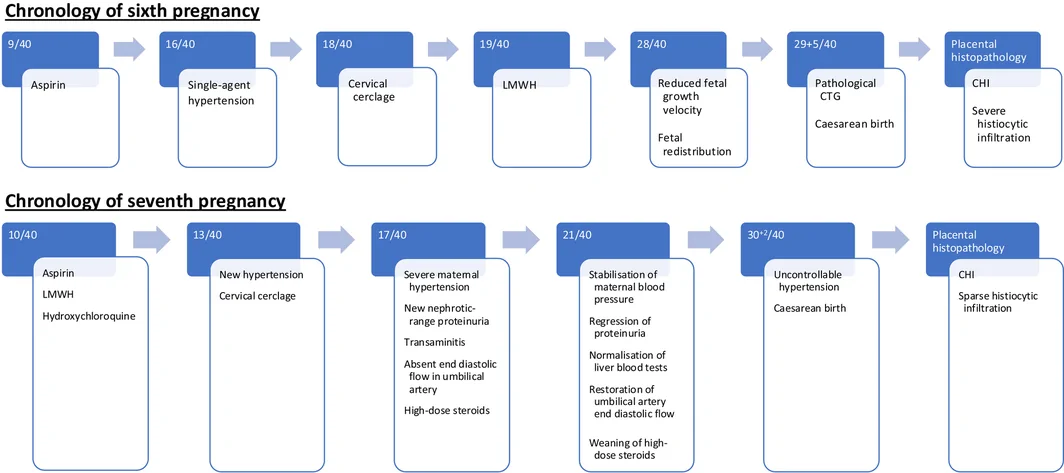
Chronology of sixth and seventh pregnancy.

Figure 2 details the chronology of clinical events in each pregnancy.

## 3 Discussion

This case demonstrates a clinical response to high dose steroids (0.5 mg/kg) in recurrent CHI presenting at 17 weeks with uteroplacental dysfunction and a maternal phenotype mimicking pre‐eclampsia with severe features. The use of high dose steroids induced stable blood pressure control, resolution of transaminitis, restoration of umbilical artery end‐diastolic blood flow, and regression of nephrotic‐range proteinuria allowing prolongation of pregnancy for an additional 13 weeks.

This case highlights the complexity of antenatal CHI diagnosis with limited data to inform management. In this case, uteroplacental dysfunction developed at 17 weeks despite early pregnancy use of low dose aspirin, prophylactic LMWH, and hydroxychloroquine. Given the service user's history and no evidence of other causes of early severe preeclampsia, the working diagnosis was recurrent CHI triggering uteroplacental insufficiency. The use of aspirin and LMWH is supported by existing literature as a strategy to improve placental function and reduce the risk of recurrent fetal loss in patients with CHI [5]. However, neither agent addresses the hypothesized maternal immune etiology.

In this case, anti‐cardiolipin antibody positivity potentially supports an autoimmune hypothesis, with antiphospholipid antibody positivity linked to CHI in other published literature [1]. Hydroxychloroquine is used in pregnancies complicated by autoimmune conditions, including CHI [6]. A systematic review looking at the effect of hydroxychloroquine on placental function has shown increased anti‐inflammatory interleukin 10 secretion and promotion of syncytiotrophoblast regeneration [7]. Although there is limited evidence of efficacy in CHI, studies have suggested that it may reduce the risk of fetal loss and improve pregnancy outcomes [8]. Retrospective case series data examining use of hydroxychloroquine (400 mg/24 h) and prednisolone (20 mg daily) showed that use of at least one immunomodulatory agent reduced histopathological severity of CHI and had a direct effect on live birth rate [9]. In this case, prednisolone was not initially chosen as a first line intervention due to risk of infection in the context of cervical incompetence and preterm birth. It is difficult to assess the efficacy of hydroxychloroquine in this case as uteroplacental dysfunction developed despite use.

Prednisolone was used in this case as a rescue therapy. Use of prophylactic prednisolone (10–20 mg daily) prior to the development of uteroplacental dysfunction is described in CHI [1, 9]. A clinical response to corticosteroid rescue therapy once uteroplacental dysfunction has developed has not previously been described in existing literature. In this case, a high prednisolone dose (0.5 mg/kg) was used with the aim of suppressing the presumed pathological maternal immune response to the placenta. Following improvement in both maternal and fetal parameters, the corticosteroid dose was weaned in a treatment regimen comparable to that of immune‐mediated solid organ transplant rejection. Autoimmune serology was not repeated after steroid initiation.

Histological examination revealed markedly less histiocytes in the intervillous spaces and overall less inflammation in the placenta of the pregnancy managed with prednisolone, compared to histology from the preceding pregnancy. CHI typically worsens in subsequent pregnancies, with a more severe phenotype in later pregnancies [10]. We hypothesize that the longer gestation at delivery and the histological improvement in placental inflammation in the later pregnancy in this case were due to the administration of corticosteroids and amelioration of CHI.

This case has shown the importance of diagnosis of CHI and the potential for high‐dose corticosteroids to ameliorate CHI allowing prolongation of pregnancy. Uniquely, high‐dose corticosteroids were used as a rescue measure, as opposed to the more commonly described prophylaxis [1, 9]. The case also highlights the crucial role of the multidisciplinary team, and careful monitoring post initiation of steroids, due to the potential for further maternal complications [11].

This is an isolated case in which high‐dose corticosteroids had a presumed therapeutic effect based on normalization of clinical parameters post steroid initiation. This corticosteroid effect needs to be reproduced in case control and/or randomized studies. Further research is needed to clarify the roles of both prophylactic and rescue immunosuppression in CHI and to optimize treatment strategies.

## Author Contributions

**Arti Dave:** methodology, writing – original draft, writing – review and editing. **Victoria Bryant:** investigation, writing – review and editing. **Manlo Kwok:** writing – review and editing. **Rebecca Allen:** writing – review and editing. **Alexander Heazell:** writing – review and editing. **Kate Wiles:** conceptualization, data curation, supervision, writing – original draft, writing – review and editing.

## Funding

The authors have nothing to report.

## Disclosure

An earlier version of this article was presented at a conference as a poster at the World Fetal Medicine Congress in 2025 in Prague, Czech Republic.

## Ethics Statement

The authors have nothing to report.

## Consent

Written and informed consent was given by the service user for publication of the clinical details and associated figures in this case report. The service user's confidentiality has been strictly maintained. CARE case report guidelines were followed and adhered to in the preparation of this case report. Informed written consent has been obtained from the patient.

## Conflicts of Interest

The authors declare no conflicts of interest.

## Supporting information


**Data S1:** CARE‐checklist‐ CHI case report.

## Data Availability

The data supporting the findings of this study are not publicly available due to the inclusion of confidential patient information and restrictions imposed by the approving institutional ethics committee and hospital guidelines. Sharing of individual‐level data could compromise participant privacy and does not comply with data protection regulations. Data may be made available from the corresponding author upon reasonable request, subject to approval by the relevant ethics committee and institution, and in accordance with applicable data protection laws.
