# Supplementary material for: High Dose Corticosteroids in Chronic Histiocytic Intervillositis Allowing Prolongation of Pregnancy: A Case Report
Source: J Obstet Gynaecol Res. 2026 Sep 7;52(9):e70478. doi: 10.1111/jog.70478 (PMC13550697; doi:10.1111/jog.70478)
Supplement: Supplementary file 1 [file JOG-52-0-s001.pdf]

CARE Checklist of information to include when writing a case report

CARE case report guidelines

(cc) BY-NC-ND

Topic	Item	Checklist item description	Reported on Line
Title	1	The diagnosis or intervention of primary focus followed by the words “case report”	Page 1, Line 1
Key Words	2	2 to 5 key words that identify diagnoses or interventions in this case report, including "case report"	Page 2, Line 22
Abstract (no references)	3a	Introduction: What is unique about this case and what does it add to the scientific literature?	Page 2, Line 6
	3b	Main symptoms and/or important clinical findings	Page 2, Line 8
	3c	The main diagnoses, therapeutic interventions, and outcomes	Page 2, Line 9
	3d	Conclusion—What is the main “take-away” lesson(s) from this case?	Page 2, Line 15
Introduction	4	One or two paragraphs summarizing why this case is unique (**may include references**)	Page 3, Line 6
Patient Information	5a	De-identified patient specific information.	Page 3, Line 25
	5b	Primary concerns and symptoms of the patient.	Page 3, Line 26
	5c	Medical, family, and psycho-social history including relevant genetic information	Page 3, Line 26
	5d	Relevant past interventions with outcomes	Page 3, Line 25
Clinical Findings	6	Describe significant physical examination (PE) and important clinical findings.	Page 3, Line 26
Timeline	7	Historical and current information from this episode of care organized as a timeline	Page 3, Line 27
Diagnostic Assessment	8a	Diagnostic testing (such as PE, laboratory testing, imaging, surveys).	Page 4, Line 1
	8b	Diagnostic challenges (such as access to testing, financial, or cultural)	Page 3, Line 29
	8c	Diagnosis (including other diagnoses considered)	Page 4, Line 10
	8d	Prognosis (such as staging in oncology) where applicable	Page 4, Line 1
Therapeutic Intervention	9a	Types of therapeutic intervention (such as pharmacologic, surgical, preventive, self-care)	Page 4, Line 31
	9b	Administration of therapeutic intervention (such as dosage, strength, duration)	Page 4, Line 31
	9c	Changes in therapeutic intervention (with rationale)	Page 4, Line 31
Follow-up and Outcomes	10a	Clinician and patient-assessed outcomes (if available)	Page 4, Line 35
	10b	Important follow-up diagnostic and other test results	Page 4, Line 40
	10c	Intervention adherence and tolerability (How was this assessed?)	Page 4, Line 32
	10d	Adverse and unanticipated events	Page 4, Line 37
Discussion	11a	A scientific discussion of the strengths AND limitations associated with this case report	Page 5, Line 24
	11b	Discussion of the relevant medical literature **with references**.	Page 5, Line 24
	11c	The scientific rationale for any conclusions (including assessment of possible causes)	Page 5, Line 41
	11d	The primary “take-away” lessons of this case report (without references) in a one paragraph conclusion	Page 6, Line 31
Patient Perspective	12	The patient should share their perspective in one to two paragraphs on the treatment(s) they received	Page 7, Line 1
Informed Consent	13	Did the patient give informed consent? Please provide if requested	Yes ☑ No ☐
